# Comprehensive bioinformatics analytics and *in vivo* validation reveal SLC31A1 as an emerging diagnostic biomarker for acute myocardial infarction

**DOI:** 10.18632/aging.205199

**Published:** 2024-05-06

**Authors:** Shujing Zhou, Longbin Wang, Xufeng Huang, Ting Wang, Yidan Tang, Ying Liu, Ming Xu

**Affiliations:** 1 Department of Clinical Veterinary Medicine, Huazhong Agricultural University, Wuhan, China; 2 Faculty of Medicine, University of Debrecen, Debrecen, Hungary; 3 Department of Cardiology, Sixth Medical Center, PLA General Hospital, Beijing, China

**Keywords:** acute myocardial infarction, bioinformatics, machine learning, cuproptosis, diagnostic biomarkers

## Abstract

Background: Globally, Acute Myocardial Infarction (AMI) is a common cause of heart failure (HF), which has been a leading cause of mortality resulting from non-communicable diseases. On the other hand, increasing evidence suggests that the role of energy production within the mitochondria strongly links to the development and progression of heart diseases, while Cuproptosis, a newly identified cell death mechanism, has not yet been comprehensively analyzed from the aspect of cardiovascular medicine.

Materials and Methods: 8 transcriptome profiles curated from the GEO database were integrated, from which a diagnostic model based on the Stacking algorithm was established. The efficacy of the model was evaluated in a multifaced manner (i.e., by Precision-Recall curve, Receiver Operative Characteristic curve, etc.). We also sequenced our animal models at the bulk RNA level and conducted qPCR and immunohistochemical staining, with which we further validated the expression of the key contributor gene to the model. Finally, we explored the immune implications of the key contributor gene.

Results: A merged machine learning model containing 4 Cuproptosis-related genes (i.e., PDHB, CDKN2A, GLS, and SLC31A1) for robust AMI diagnosis was developed, in which SLC31A1 served as the key contributor. Through *in vivo* modeling, we validated the aberrant overexpression of SLC31A1 in AMI. Besides, further transcriptome analysis revealed that its high expression was correlated with significant potential immunological implications in the infiltration of many immune cell types, especially monocyte.

Conclusions: We constructed an AMI diagnostic model based on Cuproptosis-related genes and validated the key contributor gene in animal modeling. We also analyzed the effects on the immune system for its overexpression in AMI.

## INTRODUCTION

Acute Myocardial Infarction (AMI) is one of the leading causes of death in developed countries. Globally, the incidence of this disease approaches 3 million people, posing significant challenges to global healthcare. Its etiology involves reduced coronary artery blood flow, leading to insufficient oxygen supply and subsequent myocardial ischemia. The decrease in coronary artery blood flow is multifactorial, potentially caused by the rupture of atherosclerotic plaques that lead to thrombus formation, coronary artery embolism, abuse of cocaine that causes cardiac ischemia, coronary artery dissection, and coronary vasospasm. AMI is characterized by its sudden onset, rapid progression, and grave prognosis. Despite the extensive efforts in pharmaceutical and surgical interventions, there has been limited improvement in the incidence and mortality rates associated with AMI over the past decades [1]. Within this context, emphasizing the urgency of AMI diagnosis cannot be overstated, as timely identification is crucial for patient survival.

Currently, cardiac troponin (encoded by the TNNI3 gene) and creatine kinase MB isoenzyme (encoded by the CKM gene) serve as the gold standard biomarkers for AMI diagnosis, although their specificity and sensitivity are less satisfactory [2, 3]. Furthermore, classic risk factors such as smoking, obesity, and hypertension play pivotal roles in prevention and clinical management, although they are insufficient for immediate diagnosis [4]. As such, the pursuit of more precise diagnostic methods and a comprehensive understanding of the complex pathogenesis and risk factors are crucial for enhancing AMI management and outcomes.

Fortunately, thanks to the rapid development in genetic engineering, microarray technology was advanced and applied in both clinical investigations and academic research. Some more new biomarkers with the potential to outperform the aforementioned were proposed in recent years. For example, Zhang et al. found that ARG1 might play a key role in the pathogenesis of AMI, which could be a biomarker of AMI and provide a reference for a more in-depth study [5]. Chen et al. identified TBX21 and PRF1 as novel diagnostic biomarkers and potential modulatory targets through comprehensive bioinformatic analytics [6]. Furthermore, the activation of the immune system after the occurrence of AMI is more and more aware by scientists and clinicians these days. Immune cell types including M2 macrophages, mast cells, and eosinophils have been proven to possess certain impacts on patients after AMI, providing new insights into the immune mechanisms of AMI pathogenesis [7]. Meanwhile, the introduction of artificial intelligence (AI) into the field of bioinformatics analytics improves the robustness of *in-silico* methods significantly. By combining these techniques, the discovery of even more reliable biomarkers can be expected in the foreseeable future [8].

Abnormal copper metabolism has been proven to be linked with heart ischemia for a long time [9–11]. Cuproptosis, a novel cell death mechanism induced by an intracellular imbalance of copper ions may also have an important role in this regard as copper has been proven to coordinate a variety of cellular biological processes such as lipolysis, cell proliferation, autophagy, and neural activity [12].

Inspired by the aforementioned, the present study aimed to establish a novel diagnostic model for early AMI detection based on the Cuproptosis-related gene set. In total, the AI for AMI diagnosis was constructed on the basis of 4 contributor genes, including PDHB, CDKN2A, GLS, and SLC31A1. Then, we performed bulk RNA sequencing with our *in vivo* models to assemble our own small cohort. By comparing with the public datasets, we determined the central role of SLC31A1. Furthermore, we validated the expression of SLC31A1 by a series of *in vivo* assays (i.e., quantitative real-time PCR, immunohistochemical staining, and their quantitative analyses by the ImageJ software) and explored the potential immunological implications of SLC31A1 expression.

**Figure 1:**
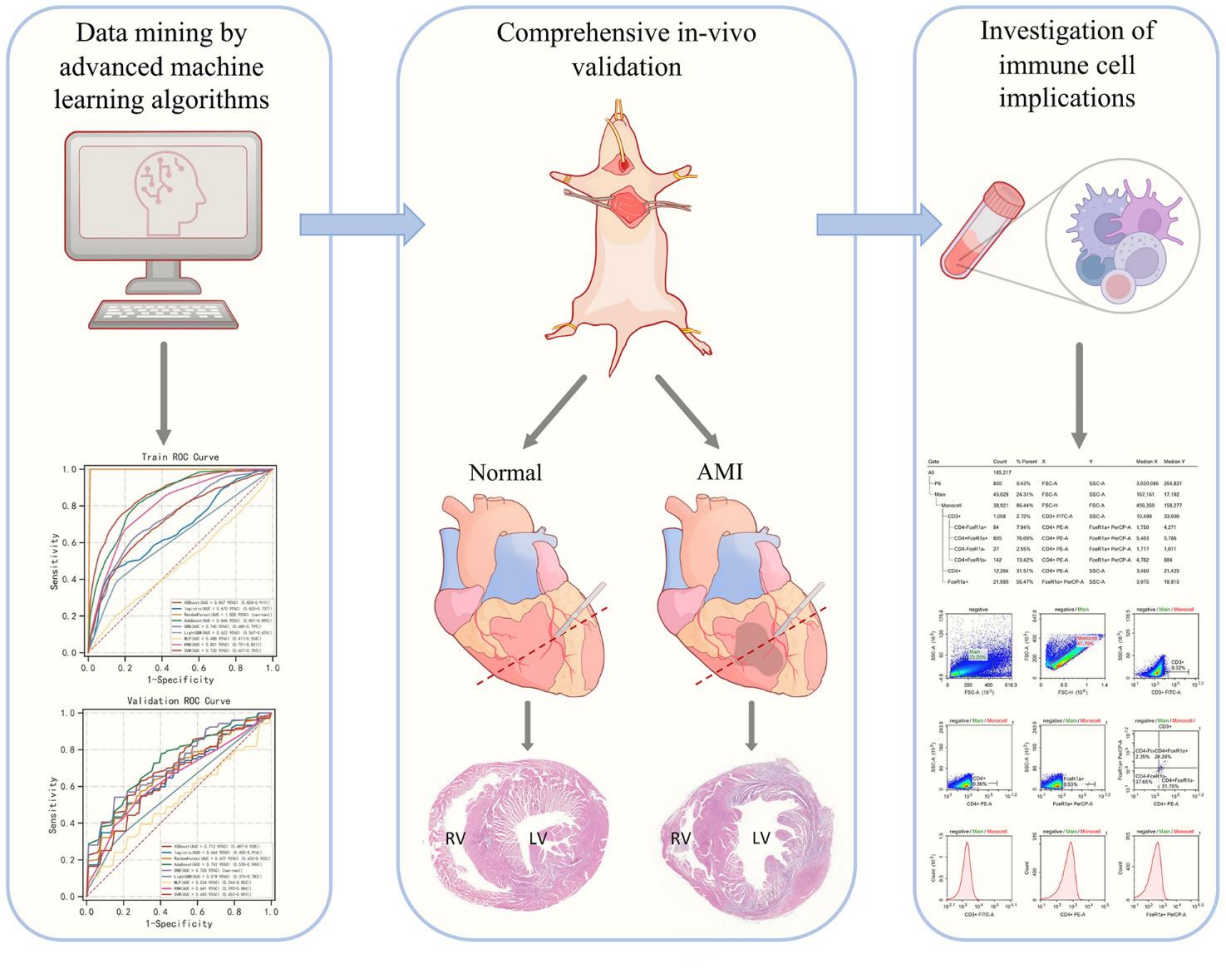
Graphical abstract of the present study.

Figure 1 demonstrates the general design of the present study.

## MATERIALS AND METHODS

### Data curation and processing

In total, 8 datasets (i.e., GSE29111, GSE60993, GSE109048, GSE29532, GSE19339, GSE48060, GSE66360, GSE97320) were curated from the GEO database (https://www.ncbi.nlm.nih.gov/geo/) [13–20]. A total of 318 samples were therefore enrolled in the present study. The normalization and calibration were done through the “Normalize Between Arrays” function of the R package, “limma”. As for the genes set involved in the present study, a total of 13 Cuproptosis-related genes, including 7 pro-Cuproptosis genes (i.e., FDX1, LIAS, LIPT1, DLD, DLAT, PDHA1, and PDHB), 3 anti-Cuproptosis genes (i.e., MTF1, GLS, and CDKN2A), and 3 copper transporter-encoding genes (i.e., ATP7A, ATP7B, and SLC31A1) [21, 22]. Of note that some genes (i.e., LIPT1, MTF1, ATP7A, and ATP7B) were not present in all the datasets and thus were not considered in the statistics. The aforementioned analyses were done online through the integrative tool set “Xsmart plateform” (https://www.xsmartanalysis.com/) by different packages embedded in the R studio software and Python (especially the Python package “sklearn”). If not specifically mentioned, the statistic test used in the analytics is the Wilcoxon rank sum test. Notably, within some figures, *, **, and *** may occur, indicative of a P-value less than 0.05, 0.01, and 0.001, respectively.

### Selection of contributor genes

To avoid linearity problems, we used not only popular linear algorithms such as LASSO but also non-linear ones like SVM-RFE, XGBoost-RFE, and Boruta algorithms [23–25]. Each algorithm gives a certain list of genes that can serve as contributor genes for the construction of diagnostic AI, but we intended to limit the number of genes involved to make it more clinically applicable. Therefore, a Venn diagram was drawn to screen the overlapping genes out. Those genes were thought as most ideal contributor genes for modeling in the next step.

### Mainstream machine learning algorithms used in the present study

According to the “no free lunch” theorem, if one machine learning algorithm outperforms the others on a specific assessment, it should sacrifice certain points on the other assessment measurements [26]. Therefore, to overcome this disadvantage, we first exhaustively tried 9 mainstream stream algorithms, including XGBoost, Logistic, Random Forest, AdaBoost, GNB, LightGBM, MLP, KNN, and SVM, to select the most suitable algorithms for this task, and then merge them by stacking method. The concept of Stacking involves first training base learners on the original data. These base learners each generate outputs based on the original data. These outputs from the multiple models are then combined to form new data, which is subsequently fed into a second-level model for fitting. This usually results in a more accurate output ultimately. In the present study, the training set and validation set contain a sample ratio of 7:3. In the modulation of stacking, the sample allocation ratio of the training set:validation set:test set is 7:2:1. All samples involved in the machine learning process were randomized first, and then allocated.

### Decision curve analysis (DCA)

Most of the time, in the case of clinical questions, false positives and false negatives are inevitable. To address this issue, DCA, which is a popular method to compare the efficacy of different predictive models under such circumstances, was used, so that the clinical benefits are maximized [27, 28].

### Calibration of predictions

To further assess the deviation between the predictive results and the reality, a calibration curve in which the predictive results were plotted against the observed reality within a randomly divided subset from the whole merged dataset. The ratio of the cases contained by this subset to the whole merged dataset is 3:10. The closer the curve is situated to the 450 ideal dash line, the more accurate prediction is given.

### Analysis of the immunological microenvironment

CIBERSORT (https://cibersort.stanford.edu/) was used to assess the abundance of various infiltrating immune cells [29, 30]. Overall, 22 immune cell types were quantified. Correlation analysis between the immune cell types and SLC31A1 was done by the Spearman method. The visualization was achieved by the R package “ggplot2”.

### Construction of *in vivo* model

All experiments were performed on male C57BL/6 J mice (6-8 weeks of age, n=6/group). Animals were housed in groups in an environment with a 12/ 12 h day/night cycle and free access to water and food.

The mice were anesthetized with isoflurane first and then opened the chest quickly. Then, the left anterior descending coronary artery was ligated together with the great cardiac vein. Immediately after the coronary artery is lapped, the heart is placed back into the chest cavity, where it is gently pressed to expel air from the chest cavity while sutures from the skin are closed. The mice were evaluated by observing their status after surgery. In the healthy control group (Sham group), the thoracic cavity was opened without ligation of the left anterior descending branch of the coronary artery. The observation indexes were the vitality and normal life of mice, and the changes in electrocardiogram before and after the operation. Finally, the mice were sacrificed by neck amputation.

### RNA sequencing and data analysis

Whole-genome gene expression analysis was performed using the heart tissues of mice from AMI and Sham groups (n=3/group) at 24h. The total RNA was extracted using Trizol (Vazyme), and cDNA samples were sequenced using a sequencing system (Novaseq 6000; Illumina). The reference Mus musculus genome and gene information were downloaded from the National Center for Biotechnology Information database. Raw reads were filtered to produce high-quality clean data. All the subsequent analyses were performed with clean data. The expression matrixes of selected genes involved in the figures were organized as tables in Supplementary Table 1.

### Quantitative real-time PCR (qPCR)

Total RNA was isolated from tissues or cells using Trizol (Vazyme), and RNA concentration and purity were measured using spectrophotometry. RNA was reverse transcribed using the PrimeScript RT reagent Kit (Vazyme) under the manufacturer’s instructions. Quantitative PCR was performed using LightCycler 96 (Roche) and SYBR Mastermix (Vazyme) in accordance with the manufacturer’s instructions. The fold difference in gene expression was 6 calculated using the 2-ΔΔ Ct method and is presented relative to Gapdh mRNA. All reactions were performed in triplicate, and specificity was monitored using melting curve analysis.

The PCR primer used in the present study was designed for the SLC31A1 gene, shown as follows:

Forward 5’-3’: GGAGAAATGGCTGGAGCTTTT

Reverse 5’-3’: CGGGCTATCTTGAGTCCTTCA

### Histological examination

For histological analysis, hearts were fixed overnight in 4% paraformaldehyde (pH 7.4), embedded in paraffin, and serially sectioned at 5-μm thickness. The sections were stained with Hematoxylin and Eosin (H&E) for routine histological examination with a light microscope. To measure collagen deposits, select sections were stained with Masson’s trichrome (MT) reagent. For each mouse, 3 random sections were quantified using ImageJ software (National Institutes of Health). Details can be checked in Supplementary Figure 1.

### Immunohistochemistry

Serial sections were deparaffinized and blocked with phosphate-buffered saline (PBS) containing 5% (v/v) normal goat serum and 1% (w/v) BSA; the sections were then incubated with anti-GLS1 (Cat. No. bs-10341R, Bioss) antibody and anti-SLC31a1 (Cat. No. bs-10773R, Bioss) overnight, followed by incubation with a secondary antibody for 1 hour at room temperature. The relative intensity of protein staining was analyzed in five random sections, chosen 40× fields for each sample and quantified using ImageJ software (National Institutes of Health). Detailed slides can be checked in Supplementary Figure 2.

### Statistical analysis used in *in vivo* experiments

The data were analyzed and graphed using GraphPad Prism 9.4.1 software and are shown as mean ± SD. The Shapiro–Wilk-test was used to detect the normal distribution. Student’s t-test or one-way ANOVA followed by Tukey’s post-hoc test was used for statistical analysis as appropriate. For the Kaplan–Meier survival plots, statistical significance was measured by the log-rank (Mantel–Cox) test. A P-value < 0.05 was deemed statistically significant. All experiments were repeated independently 3 times.

## RESULTS

### Data normalization and the selection of contributor genes for machine learning

**Figure 2:**
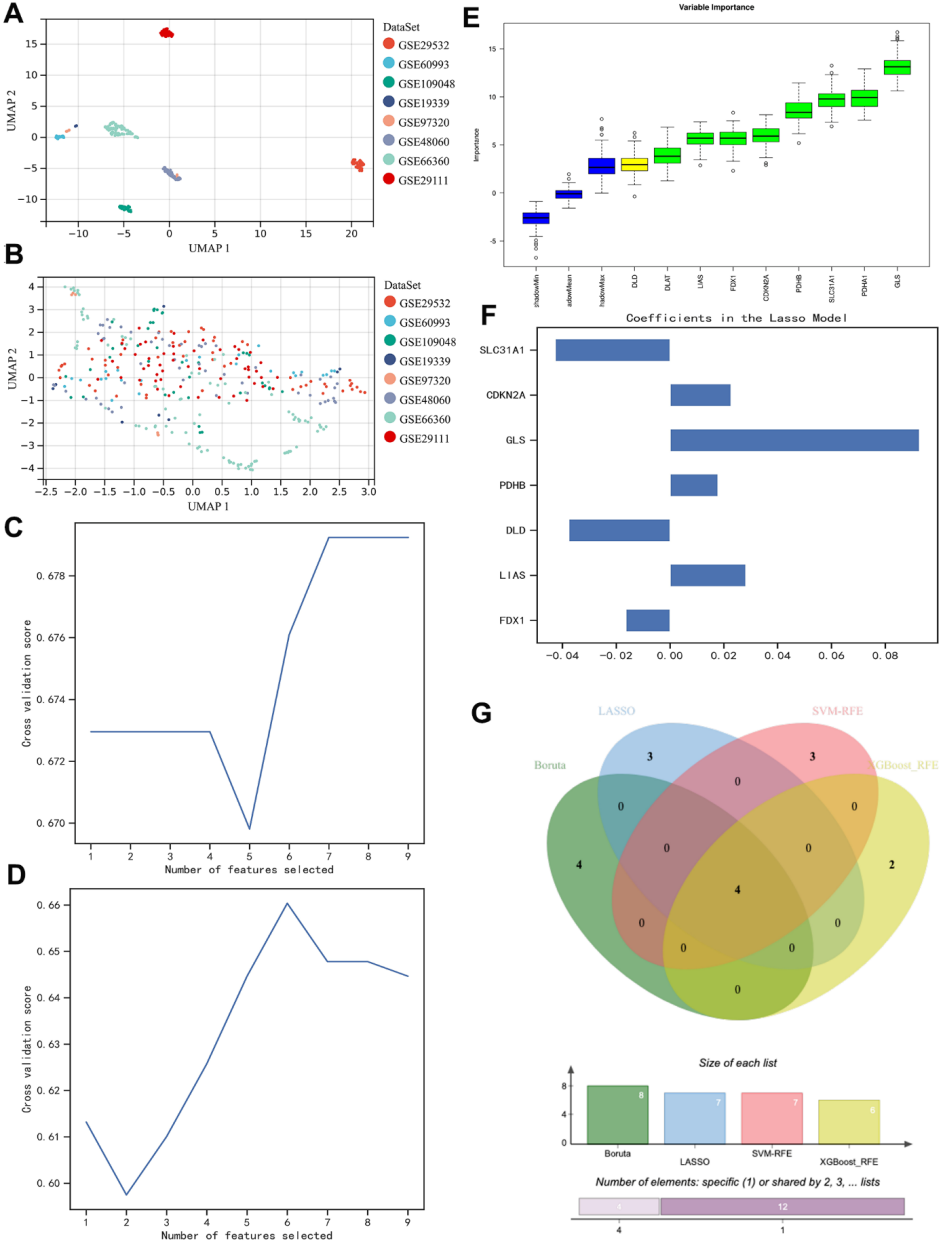
**Homogenization of different samples from various datasets and selection of the contributor genes for the construction of AI diagnostic predictor.** (**A**, **B**) UMAP plot visualizes the sample distribution. As shown, originally the samples were fairly separated (**A**), but they were very well homogenous after normalization (**B**). (**C**–**F**) Determination of suitable contributor genes by SVM-RFE, XGBoost-RFE, Boruta, and LASSO, respectively. (**G**) Venn diagram demonstrated the overlapping genes. 4 genes (i.e., PDHB, CDKN2A, GLS, and SLC31A1) were finally selected.

The expression matrixes of the 8 GEO datasets were normalized with a baseline correction and then merged. To see if the samples were homogenized, we visualized them in the form of UMAP. As shown in the figure, the samples involved in the present study were well-mixed with one another, indicating a good homogeneity (Figure 2A, 2B).

Then, we went on with contributor gene selection. Through the SVM-RFE algorithm, 7 genes (i.e., FDX1, DLD, PDHA1, PDHB, GLS, CDKN2A, and SLC31A1) were thought to be the most suitable for the following AI construction (Figure 2C), while the results of XGBoost-RFE algorithm recommended LIAS, PDHA1, PDHB, GLS, CDKN2A, and SLC31A1 (Figure 2D). In the Boruta algorithm, FDX1, DLAT, LIAS, PDHA1, PDHB, GLS, CDKN2A, and SLC31A1 were filtered out and ranked with specific weights of importance (Figure 2E). Besides the aforementioned non-linear algorithms, the regularly used linear algorithm in the field of bioinformatics, LASSO, was also employed in the present study, through which we found FDX1, LIAS, DLD, PDHB, GLS, CDKN2A, and SLC31A1 were the strongest candidates (Figure 2F). To sum up, we used a Venn diagram to intersect the overlapping genes (Figure 2G), so that the contributor genes for AI construction could be determined. As a result, we decided to use PDHB, CDKN2A, GLS, and SLC31A1 in the rest of the study.

### Selecting the best algorithms

**Figure 3:**
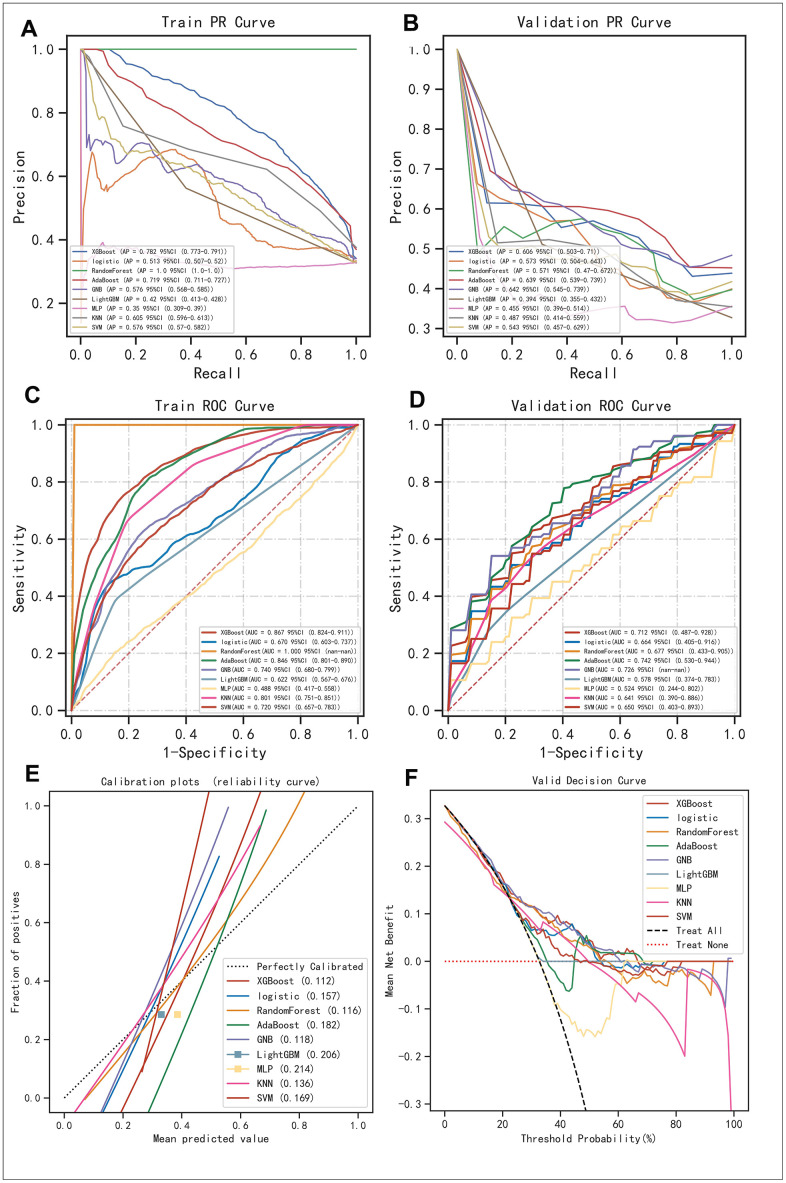
**Evaluation of the robustness of the 9 mainstream machine algorithms to identify the best ones for AI construction.** (**A**, **B**) Precision-Recall (PR) curve in the training set and validation set, respectively. (**C**, **D**) Receiver Operative Characteristic (ROC) curve performed in the training set and validation set, respectively. (**E**) Calibration curve demonstrating the bias between predictive values and realistic values for the machine learning algorithms involved in the present study. (**F**) DCA for the machine learning algorithms involved in the present study.

We exhaustively went through 9 mainstream machine learning algorithms (i.e., XGBoost, Logistic, Random Forest, AdaBoost, GNB, LightGBM, MLP, KNN, and SVM) to identify the ideal machine learning algorithms for this task. Consequently, we found that regarding the Precision-Recall rate (i.e., the so-called “PR” annotated in the figure), XGBoost, AdaBoost, and GBN were the most superior algorithms (Figure 3A, 3B), which was further confirmed by the value of Area under Curve (AUC) on the Receiver Operative Characteristic (ROC) curve (Figure 3C, 3D). Although the AdaBoost algorithm possessed a little bit higher bias in the calibration curve (i.e., 0.182) than that of the Random Forest algorithm (i.e., 0.116), and was located comparatively lower in the plot of DCA, comprehensively considering, we believed XGBoost, AdaBoost, and GBN were the most ideal options for further AI modulation (Figure 3E, 3F).

### The stacking of machine learning algorithms outperformed the gold standard biomarkers in AMI

**Figure 4:**
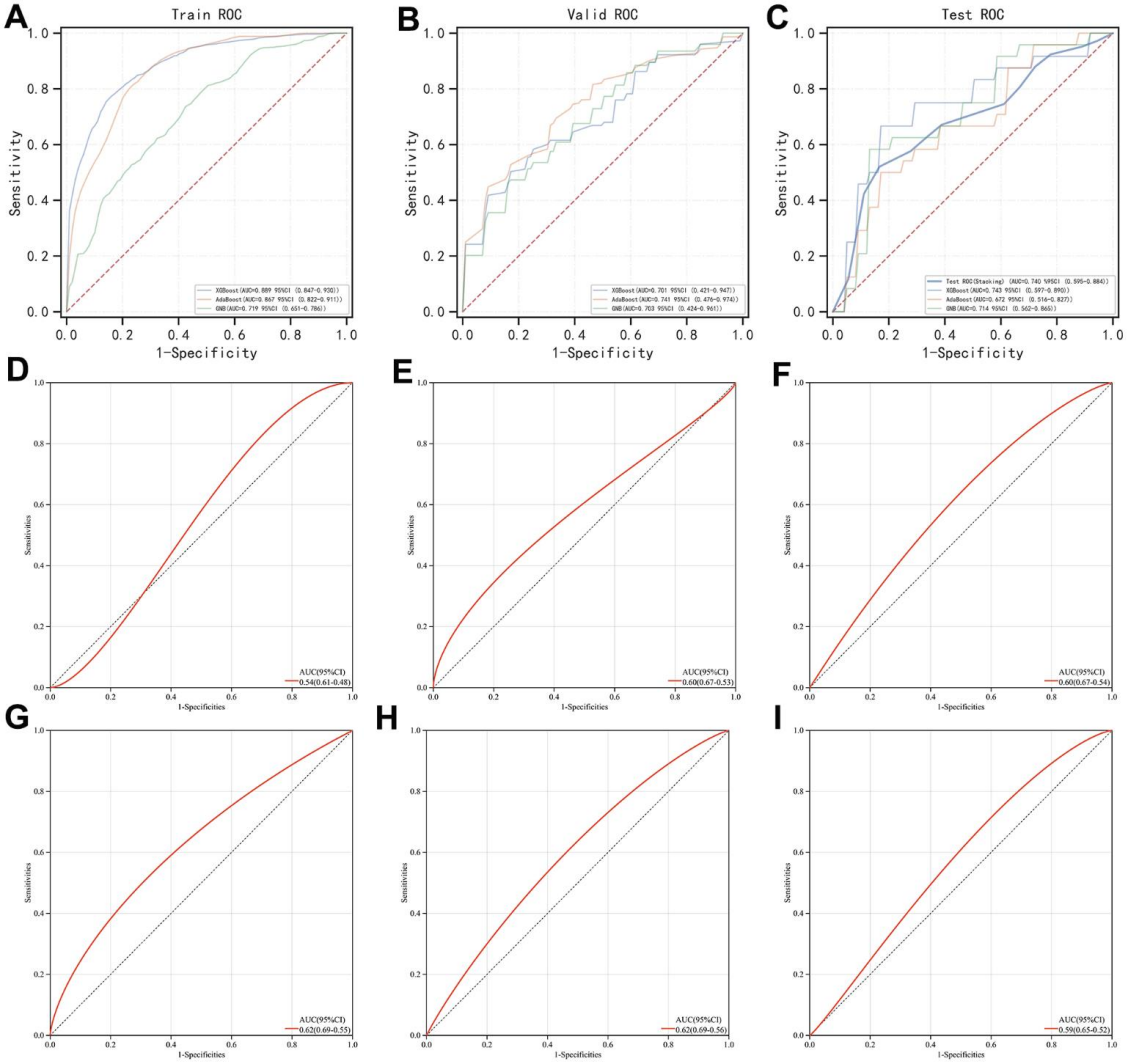
**Evaluation of the stacking-based AI predictor for AMI diagnosis and comparison of its efficacy with individual genes involved (i.e., PDHB, CDKN2A, GLS, and SLC31A1) and gold standard biomarkers (i.e., TNNI3 and CKM).** (**A**–**C**) ROC curve of the stacking-based AI predictor in the training set, validation set, and test set, respectively. (**D**–**G**) ROC curve of PDHB, CDKN2A, GLS, and SLC31A1, respectively. (**H**, **I**) ROC curve of TNNI3 and CKM, respectively.

As previously described, we found that XGBoost, AdaBoost, and GBN were seemingly the best options for AI modulation as they possessed relatively high PR-AUC and ROC-AUC values. While in terms of the stacking method, usually 2 layers of classifying logic are set, and the difference between the 2 layers is positively associated with the final predictive outcome. Therefore, besides placing XGBoost, AdaBoost, and GBN into the first layer, we used MLP which performed most distinctly from these algorithms as a second layer. Subsequently, we found the merged version behaved satisfying predictions with a ROC-AUC value over 0.7 in not only the training set but also the validation set and even the test set respectively, showcasing its outstanding calculation power (Figure 4A–4C). Meanwhile, we examined the individual predictive ability of each contributor gene, finding that when using these genes as diagnostic biomarkers solely, the outcomes were fall out of our expectations as PDHB (Figure 4E), SLC31A1 (Figure 4F), and GLS (Figure 4G) possessed a ROC-AUC value of around 0.6 respectively and CDKN2A (Figure 4D) had only reached 0.54. Gold standard biomarkers (i.e., TNNI3 and CKM) on the other hand were not playing well either, as they were scored with a ROC-AUC value of 0.62 and 0.59, respectively (Figure 4H, 4I). Overall, by combing the 4 contributor genes as a genetic signature, with the proper assistance of AI, the early diagnosis of AMI would likely reach a new level.

### *In vivo* validation of SLC31A1 expression

**Figure 5:**
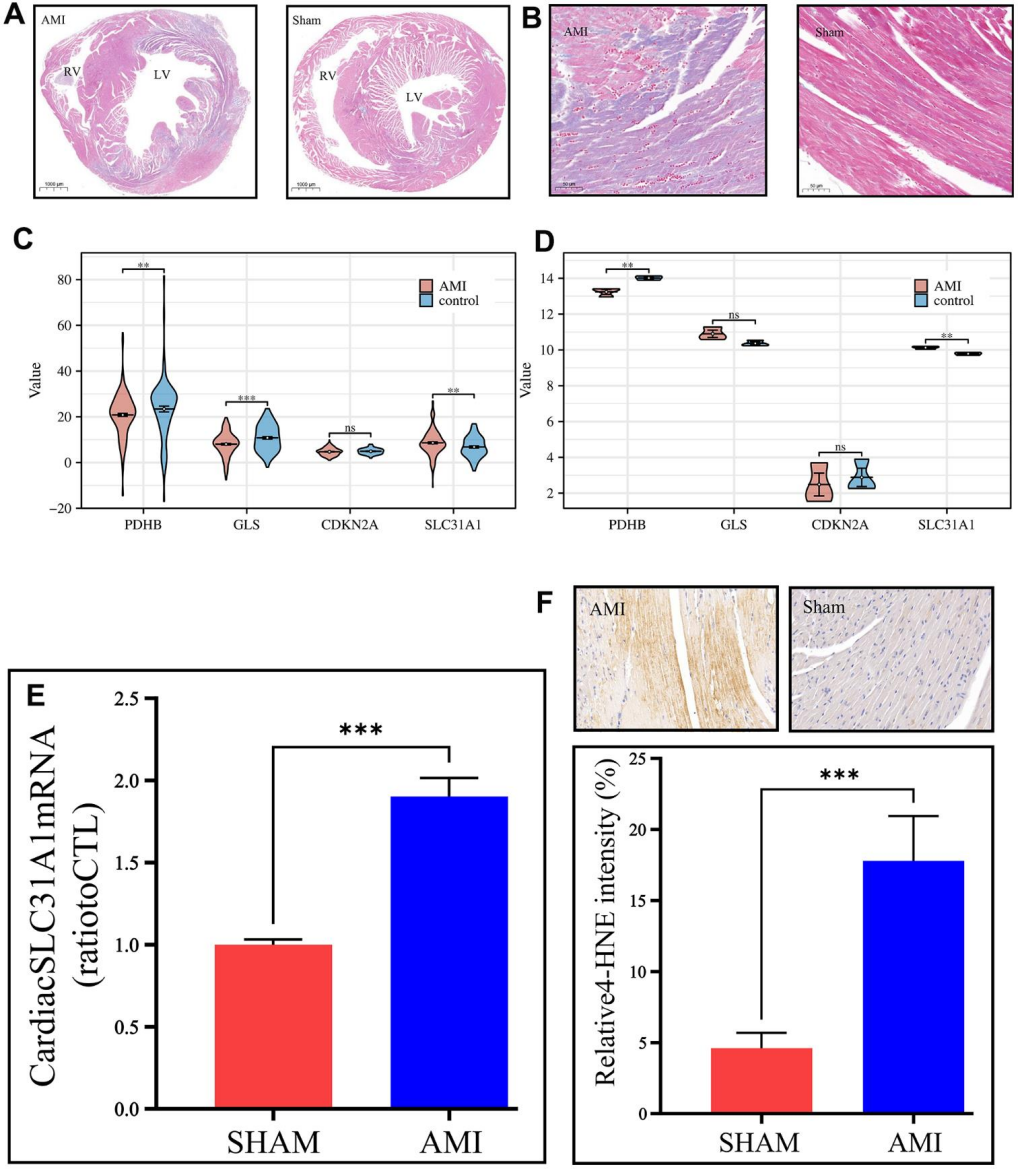
***In vivo* validation of the expression of the contributor genes, with a focus on the SLC31A1 gene.** (**A**) Masson staining slides on a macroscopic view, demonstrating the coronal section of the mice’s hearts. The left panel is the AMI sample, and the right panel is the Sham sample. RV: right ventricle, LV: left ventricle. (**B**) Masson staining slides of the coronary heart section of the AMI group and Sham group under magnification. The purplish color indicates hypoxia, thus the area of AMI. The deeper the color, the more severe infarction. (**C**, **D**) Expression analysis of the contributor genes in the form of violin plots in the merged GEO dataset and our own bulk RNA sequencing cohort, respectively. (**E**) qPCR results of the SLC31A1 gene expression. (**F**) Upper panel: immunohistochemical staining of the SLC31A1 protein in the AMI group and Sham group. The more brownish color, the more abundant the SLC31A1 protein. Lower panel: quantitative analysis of the immunohistochemical staining for the SLC31A1 protein in the AMI group and Sham group in the manner of bar plot.

We modeled the mice with surgical techniques described in the methodology section and validated them from histological slides with Masson staining. Representative images were exhibited within the figure. In a nutshell, we compared the AMI group and the Sham group macroscopically (Figure 5A) and under magnification (Figure 5B), both demonstrating significant differences regarding the color of staining. In the AMI group, within the region of infarction, the myocardium was in much deeper purplish, indicating a significant pathological status, while in the Sham group, normal myocardium could be clearly observed in a healthy red color.

In the GEO datasets, we tested the expression of the contributor genes in the manner of violin plots. It was found that except for the CDKN2A gene, the rest genes had very high statistical significance regarding differential expression (Figure 5C). However, it was found that in our own bulk sequencing cohort, merely the SLC31A1 gene was granted statistical significance, and the other genes possessed P-values over 0.05, although meanwhile, the mean differences were apparent (Figure 5D). As such, we continued the *in vivo* studies focusing on the SLC31A1 gene. Through the qPCR, the expression of the SLC31A1 gene was determined at the mRNA level in the AMI group and Sham groups. It was found that the SLC31A1 gene was expressed at a relatively higher level in the AMI group (Figure 5E), which supported our computational results. At the protein level, we immunohistochemically stained the samples to visualize the abundance of SLC31A1 protein (Figure 5F). Subsequently, we found the SLC31A1 protein was much more abundant in the AMI group than that in the Sham group.

### SLC31A1 expression was potentially positively correlated with monocyte infiltration

**Figure 6:**
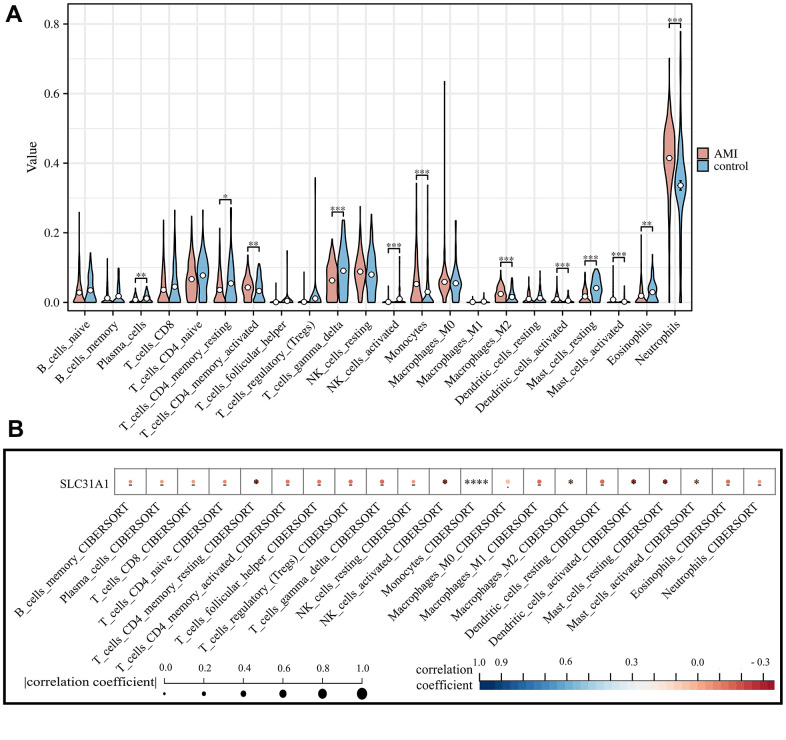
**Assessment of the infiltration of various immune cell types in both AMI and healthy control and their correlation with SLC31A1 expression.** (**A**) Violin plot demonstrating the comparison of abundance of infiltrating immune cell types in the AMI group and healthy control. (**B**) Spearman correlation analysis between the SLC31A1 expression and diverse infiltrating immune cell types.

We first obtained the abundance of various infiltrating immune cells in AMI using the CIBERSORT algorithm. There are significant differences in multiple immune cell types in AMI in comparison with the healthy control, including plasma cells, CD4+ memory T cells, Gamma-Delta T cells, activated NK cells, monocytes, macrophage M2, activated dendritic cells, mast cells, eosinophils, and neutrophils (Figure 6A). Such results were cross-validated with previous studies which pointed out that the infarction status of the myocardium might trigger a diverse immune cell activation in which there was a sophisticated interplay [31–33]. We further analyzed the association between the SLC31A1 expression and cellular immunity. We found a significant association between SLC31A1 and most of the immune cell types, especially monocytes, with a synchronizing trend in Figure 6A, hinting at the potentially central role of SLC31A1 among the contributor genes in AMI from the immunological aspect (Figure 6B).

## DISCUSSION

Cardiovascular diseases as one of the most serious diseases worldwide, especially AMI, have been affecting millions of patients yearly and can recur in more than half of the population [34, 35]. Regardless of the rapid advancements in the healthcare industry, there have been limited improvements in morbidity and mortality from AMI over the past few decades, particularly in young ages [36]. For a long time, cardiac troponin and creatine kinase-MB isoenzyme are viewed as gold standard biomarkers for AMI diagnosis [37–39], but their real effectiveness and sensitivity are questionable. In the present study, their predictive performance in our merged GEO dataset was indeed less satisfying. Identifying new biomarkers for accurate and robust AMI diagnosis remains in demand.

In 2022, Tsvetkov and colleagues discovered Cuproptosis, a novel cellular demise pathway contingent upon mitochondrial respiration [12]. It stands distinct from apoptosis by its unique reliance on copper and mitochondria interaction, meanwhile, unlike ferroptosis (also a result of imbalanced ion hemostasis), which involves iron-dependent lipid peroxidation, Cuproptosis emphasizes copper homeostasis disruption, unveiling a novel avenue in understanding cellular demise with implications beyond classical apoptotic pathways. Subsequently, extensive research has delved into its implications within the realm of oncology [40–42], although there remains a conspicuous dearth of studies investigating its pertinence to non-neoplastic conditions.

On the other hand, the wide use of high-throughput sequencing technique generated a wealth of big data that is being analyzed again and again to further explore the pathomechanisms of various diseases. However, mining valuable knowledge from such overwhelming amounts of data is a challenging and vital task in modern medicine. In this regard, machine learning, a successful methodology to extract valuable knowledge under this background, has been applied in order to identify novel therapeutic targets and optimize current treatment strategies, making early detection of the disease of interest and predictions of the corresponding prognosis, and so forth more precise and efficient.

Our study is the first to employ up to nine mainstream machine learning algorithms, including XGBoost, Logistic, Random Forest, AdaBoost, GNB, LightGBM, MLP, KNN, and SVM, as well as stacking methods to create a powerful AI for AMI diagnosis. By using these methods, we identified XGBoost, AdaBoost, GBN, and MLP as the most suitable algorithms for the task. Using the stacking method, we integrated XGBoost, AdaBoost, and GBN into the first layer of the AI's logic chain, while MLP served as the second layer. This resulted in a highly robust AI with superior predictive power compared to any of the single machine learning algorithms, the single contributor genes to the AI, and even the gold standard biomarkers (i.e., TNNI3 and CKM). In regard to the evaluation of all the aforementioned, we mainly made it based on the AUC value. In general, a model that has an AUC of 0.5 does no better than random guessing, and an AUC value over 0.7 is usually seen as a good value. By building the highly accurate model for AMI diagnosis, we hope that with the help of our model, someday physicians can estimate reliably the risk of AMI for patients under suspicion, by simply testing the expression of the contributor genes of the model.

Among the genes contributing to AMI (PDHB, CDKN2A, GLS, and SLC31A1), SLC31A1 was found to be of particular importance. In fact, SLC31A1 is a gene that encodes for the copper transporter protein 1 (CTR1) which plays a crucial role in copper homeostasis in cells. CTR1 is responsible for the uptake of copper from the extracellular environment and its transport into cells. The protein is particularly important in cells that require copper for their function, such as those involved in oxidative phosphorylation, iron metabolism, and neurotransmitter synthesis. From this end, it is somehow reasonable that SLC31A1 may impact significantly in AMI. To validate our hypothesis, we carried out qPCR, western blot, and immunohistochemical staining in *in vivo* models. The results showed that SLC31A1 was aberrantly overexpressed in cases of AMI, underscoring its potential as a diagnostic biomarker. This finding supported the previous works done by Zheng et al. and Wang et al. in which variations in the SLC31A1 gene have been associated with an increased risk of AMI and decreased CTR1 expression has been observed in rat hearts after myocardial infarction [43, 44].

Accumulated evidence had suggested that immune cells might play a critical role in the development and progression of AMI [45–48]. In particular, the infiltration of monocytes in the myocardium has been implicated in the development of ventricular remodeling and the potentially resulting heart failure [49–51]. Therefore, we were also interested in the correlation between SLC31A1 expression and various immune cell types. It turned out that there was a potentially positive association between SLC31A1 expression and the infiltration of many immune cell populations, especially monocyte. In fact, monocytes have been proven to be the versatile cells of the innate immune system, indispensable in the initial inflammatory response to injuries and subsequent wound healing processes in many tissues, including the heart [52]. As such, these findings highlighted the possible central role of SLC31A1 expression in AMI’s immune landscape and its probability in improving heart tissue recovery in the post-AMI scenario.

## CONCLUSIONS

In summary, the present study established a novel diagnostic model for early AMI detection based on the Cuproptosis-related gene set, identifying the central role of SLC31A1, and validated the aberrant overexpression of SLC31A1 in *in vivo* assays, exploring its potential immunological implications, sharing new perspective toward alternative AMI biomarker development.

## Supplementary Material

Supplementary Figures

Supplementary Table 1
