# Supplementary material for: Comprehensive bioinformatics analytics and in vivo validation reveal SLC31A1 as an emerging diagnostic biomarker for acute myocardial infarction
Source: Aging (Albany NY). 2024 May 6;16(9):8361–77. doi: 10.18632/aging.205199 (PMC11132003; doi:10.18632/aging.205199)
Supplement: Supplementary Figures [file aging-16-205199-s001.pdf]

SUPPLEMENTARY FIGURES

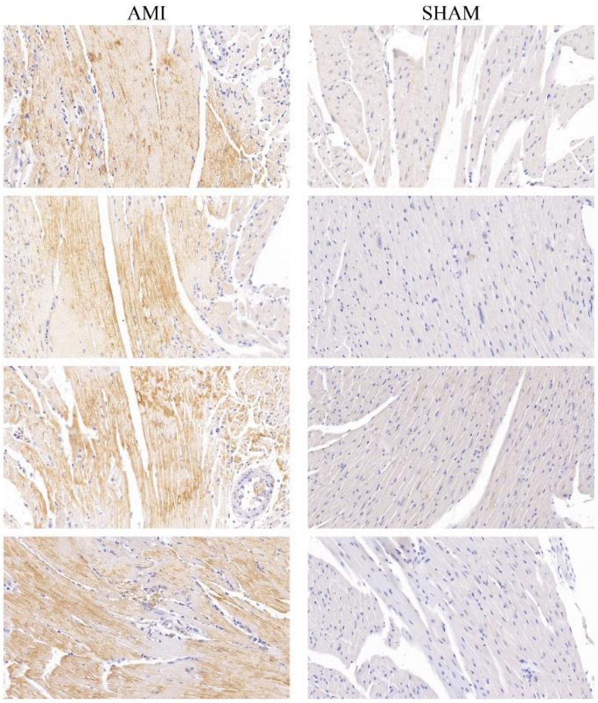

Supplementary Figure 1. Supplementary images of Masson staining slides.

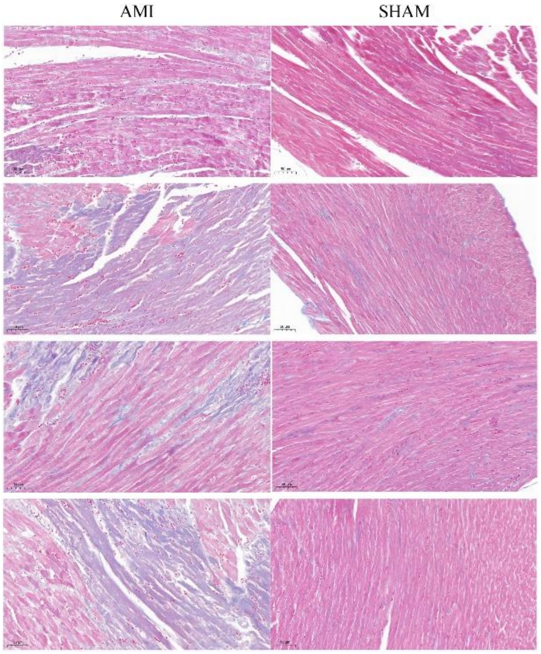

Supplementary Figure 2. IHC slides.
